# The Removal of a Foreign Body from the Duct of Wharton

**Published:** 1848-04

**Authors:** H. F. Campbell

**Affiliations:** Demonstrator of Anatomy in the Medical College of Georgia


*The Removal of a Foreign Body from the Duct of Wharton.* By H. F. Campbell, M. D., Demonstrator of Anatomy in the Medical College of Georgia.—The novelty of the following case will be seen to warrant its publication.

Of foreign bodies in the duct of Wharton, so far as we know, there is on record but one case, which is that presented by M. Robert to the Anatomical Society of Paris, and which indeed has been considered quite remarkable. It occurred in the person of a shoemaker, in which case the orifice had been found to admit a piece of hog's bristle which subsequently became the nucleus of salivary calculus.

Case. Julia, a nurse, aged 14 years, while engaged at work, with a pin in her mouth, felt pain under the tongue, and endeavored to remove the pin, but on feeling for it could only find the *point* protruding at the side of the frænum linguæ. Her efforts to extract it by the point caused it entirely to disappear: becoming alarmed, she called for assistance. On examination, there could not be seen the least trace of any foreign body whatever: she said that "the pin was under her tongue, and had gotten into the flesh *head-foremost.*" It gave her no pain, except when disturbed with the fingers; the orifice of the Whartonian duct was patulous and some saliva was flowing from it. On applying the finger to the floor of the mouth the pin could easily be felt near to the base of the lower jaw—though from the distance to which the head had proceeded towards the cœcal extremities of this duct, it was impossible to protrude it by applying pressure from behind, and further, from the handling to which the parts had been subjected, the point had been pushed out of the direction by which it entered, and having pierced the side of the duct was resting on the alveolar process. It was very moveable, and receded on the slightest pressure.

Failing of its removal by manipulation, the following method was adopted: Its exact situation being ascertained, the object together with the parts surrounding it was seized by the forefinger of the left hand in the mouth and the thumb in the digastric region, and pressed outward against the inner surface of the lower jaw under the alveolar projection: a tenaculum was then introduced from within outward through the mucous membrane, (avoiding the situation of the gustatory nerve which near this place crosses the duct,) so as to enclose the duct and hold the pin fixed; on elevating the tenaculum, the point of the pin became prominent about three lines posterior to the orifice of the duct. The mucous membrane and coats of the duct being cut through with a scalpel, the pin was removed with the dressing forceps by the *point* which protruded through the opening of the incision. A copious discharge of saliva followed its removal. The incision healed rapidly, and the patient recovered without any trouble. The pin was 1¼ inches in length, and of a proportionate thickness.

